# Investigation of Carriers of *Salmonella* and Other Hydrogen Sulphide-Positive Bacteria in the Digestive Content of Fish from the Atlantic Area of Macaronesia: A Comparative Study of Identification by API Gallery and MALDI-TOF MS

**DOI:** 10.3390/ani14223247

**Published:** 2024-11-12

**Authors:** Inmaculada Rosario Medina, Marco Antonio Suárez Benítez, María del Mar Ojeda-Vargas, Kiara Gallo, Daniel Padilla Castillo, Miguel Batista-Arteaga, Soraya Déniz Suárez, Esther Licia Díaz Rodríguez, Begoña Acosta-Hernández

**Affiliations:** 1 Departamento de Patología Animal, Facultad de Veterinaria, Universidad de Las Palmas de Gran Canaria, 35413 Arucas, Spain; marco.suarez107@alu.ulpgc.es (M.A.S.B.); kiara.gallo101@alu.ulpgc.es (K.G.); daniel.padilla@ulpgc.es (D.P.C.); miguel.batista@ulpgc.es (M.B.-A.); soraya.deniz@ulpgc.es (S.D.S.); esther.diaz@ulpgc.es (E.L.D.R.); 2 Instituto Universitario de Sanidad Animal (IUSA), Facultad de Veterinaria, Universidad de Las Palmas de Gran Canaria, 35413 Arucas, Spain; 3 Servicio de Microbiología, Complejo Hospitalario Universitario Insular Materno Infantil de Las Palmas de Gran Canaria, 35016 Las Palmas, Spain; mar.ojeda@ulpgc.es; 4 Departamento de Ciencias Clínicas, Facultad de Ciencias de la Salud, Universidad de Las Palmas de Gran Canaria, 35016 Las Palmas, Spain; 5 Instituto Universitario de Investigaciones Biomédicas y Sanitarias (iUIBS), Universidad de Las Palmas de Gran Canaria, 35413 Arucas, Spain

**Keywords:** fish carriers, pathogenic microorganisms, salmonellosis, *Enterobacter*, *Shewanella*, *Vibrio*, health risk

## Abstract

*Salmonella* spp. are known pathogens in fish, with their presence potentially resulting from the contamination of the aquatic environment or improper handling. Accurate bacterial identification is crucial across various fields, including medicine, microbiology, and the food industry, and thus a range of techniques are available for this purpose. In this study, *Salmonella* spp. and other hydrogen sulphide-positive bacteria were investigated in the digestive contents of fish destined for consumption from the Atlantic area of Macaronesia. Two identification techniques were compared: the traditional API method and the MALDI-TOF MS technique. For the identification of *Salmonella* spp. carriers, 59 samples were processed following ISO 6579–1:2017. A total of 47 strains of Gram-negative bacilli were obtained. No *Salmonella* spp. isolates were detected. The most frequent genus was *Enterobacter* (76.50%), followed by *Shewanella* (10.63%). The MALDI-TOF MS technique showed a high concordance with the API technique, with 72.34% concordance at the species level. Both techniques demonstrated a high degree of concordance in the identification of *Enterobacter cloacae*, with 87.23% genus-level concordance and 12.76% non-concordant identifications. This study highlights the limitations of the API technique and the speed and precision of MALDI-TOF MS. The identified bacteria could pose a health risk to humans.

## Simple Summary

This study addresses the issue of the detection of harmful bacteria (*Salmonella* and other hydrogen sulphide-positive bacteria) present in fish for consumption, which may arise from contaminated water or improper handling. The aim is to compare two methods for identifying these bacteria in the digestive content of fish from the Macaronesian region. A total of 59 samples were analysed. *Salmonella* was not detected, but other bacteria were identified (47), the most common being *Enterobacter*. This study revealed that the new MALDI-TOF MS method is faster and more accurate than the traditional API method, showing high concordance in the identification of bacteria. These findings are important because they highlight the limitations of older techniques and emphasise the importance of using more effective methods to ensure food safety. By improving the identification of potentially harmful bacteria, this research can help protect public health and improve safety standards in the food industry.

## 1. Introduction

The biodiversity of the sea is remarkable, comprising up to 28,000 identified fish species in addition to molluscs, crustaceans, echinoderms, cetaceans, and marine mammals, among others. A small fraction of these are commercially exploited through capture, processing, and sale to the public. Given this context, research is of vital importance to determine the presence of pathogenic microorganisms that could pose a public health risk, particularly in fish of marine origin marketed for consumption. These animals can transmit diseases when consumed, resulting in death in severe cases [1] such as salmonellosis.

*Salmonella* spp., the pathogen of focus in the present study, is a biological contaminant reported in fish, often originating from contaminated water or improper handling [2]. The ability of this bacteria to live in soil and water and its transfer to fish, highlights the need to provide information on how contamination occurs and the possible ways in which it spreads. This is essential to be able to identify the source of the bacteria in fish markets [3]. *Salmonella* belongs to the family *Enterobacteriaceae* and comprises numerous serotypes. While some serotypes only affect animals, posing an animal health problem, others can affect both humans and animals, presenting a public health issue and being the main cause of most foodborne infections [4]. Recent studies have shown the presence of this pathogen in the digestive tract of fish along with *Escherichia coli*, another enterobacterium, with prevalence rates of approximately 23% and 22%, respectively [5]. Thus, certain strains of *Escherichia coli*, specifically *E. coli* = 157: H7, can cause severe diseases such as haemorrhagic colitis and are acquired through contaminated food and water [6]. *Salmonella enterica* serotype *Enteritidis* and *Salmonella enterica* serotype *Typhimurium* can cause salmonellosis, leading to gastroenteritis and which sometimes endangers human health due to complications. Infection by both of these enterobacteria can indicate faecal contamination of food and water [7].

Other pathogenic bacteria that can be found in the aquatic environment and that can cause public health problems are described, such as *Vibrio* spp., *Campylobacter* spp., and *Staphylococcus aureus* among others. Within *Vibrio* spp., the role of *Vibrio cholerae* stands out as a Gram-negative bacterium responsible for cholera, a disease that causes profuse watery diarrhoea [8], and *V. vulnificus* and *V. parahaemolyticus* that cause gastroenteritis, septicaemia, and wound infections in humans [9]. On the other hand, other bacteria such as *Campylobacter jejuni* and *Campylobacter coli* are major causes of intestinal infections globally, leading to diseases such as colitis. Infection can occur through contaminated wastewater and food [10]. Lastly, *Staphylococcus aureus* can be found in water contaminated with animal and human waste, subsequently contaminating aquatic organisms and producing harmful toxins that cause food poisoning [11]. Further, others zoonotic water-associated bacterial pathogens like *Aeromonas* and *Plesiomonas* should be considered [12,13].

Given these health risks, correct bacterial identification is fundamental for public health, food safety, and scientific advancement [14]. Wastewater plays a significant role in this context because it can introduce pathogenic bacteria into the aquatic environment, contaminating the organisms that inhabit it. This can pose a significant public health risk from the consumption of fish contaminated with microorganisms originating from the microbial load of water [11]. Thus, the interconnectedness of environmental, animal and human health warrants a One Health approach to fish consumption.

The lack of economic resources in many countries presents a challenge for efficient wastewater management. Human activities such as industrialisation, agriculture, urbanisation, and improper waste disposal have made water pollution a constant and widespread issue [15]. The correct identification of these bacteria is necessary, and microbiology uses different methods to achieve this. These methods can be classified into phenotypic, molecular, and proteomic methods [14].

Phenotypic methods identify observable characteristics of bacteria (such as shape, size, and colour after staining) at the microscopic level and their response to nutritional requirements and biochemical tests. Biochemical identification can be performed using either manual or automated commercial galleries [16,17]. Manual commercial systems include the Analytical Profile Index (API, bioMerieux), Enterotube (BBL), Oxi/Ferm Tube (BD), RapID and MicroID systems (Remel), and biochemical identification systems (Microgen), among others. These kits determine the metabolic characteristics of microorganisms. Automated systems include MicroScan, Vitek, ATB, Pasco, Wider, Phoenix, and others. These systems provide reliable microorganism identification [14].

Molecular and genotypic methods analyse genetic material and are often used as alternatives or complements to phenotypic methods. These methods include DNA sequencing, with 16S rRNA sequencing being widely used. Other techniques include a polymerase chain reaction (PCR) to identify and amplify specific DNA sequences as well as other specific hybridisation sequences, such as 16S IT or marker sequences, for some bacterial genera. Proteomic methods analyse proteins, using techniques such as gel electrophoresis and mass spectrometry, with matrix-assisted laser desorption/ionisation time-of-flight mass spectrometry (MALDI-TOF MS) being particularly notable [14]. The MALDI-TOF MS technique has existed for many years but was not used for pathogen identification until the early 2000s [18].

MALDI-TOF MS offers numerous advantages, including broad application across various microorganisms, high efficiency, significantly faster results than provided by conventional methods that take 24–48 h, precision, lower cost, and more. The speed of microorganism identification makes MALDI-TOF MS a valuable tool in fields such as medical diagnostics, biological defence, environmental surveillance, and food quality control, however, the accuracy of MALDI-TOF MS results is highly dependent on the size of the database (limiting factor). It provides a clear alternative to traditional molecular and biochemical identification methods [19].

Despite the advantages of MALDI-TOF MS, the equipment is extremely expensive, making it difficult for laboratories to afford. This has led to the continued use of conventional methods such as biochemical activity tests and observations of bacterial morphology and growth characteristics. However, phenotypic identification has considerable limitations, such as differentiating closely related microorganisms or those with atypical phenotypes, which can lead to incorrect identifications [20]. Many test interpretations are visual, affecting final identification [21]. Other issues include inaccuracies in identification databases [22]. Despite these drawbacks, phenotypic tests based on commercial identification systems are still widely used in laboratories [21]. Nonetheless, bacterial identification using molecular techniques and MALDI-TOF MS remains promising [23].

Based on this background, the objective of this study was to detect *Salmonella* carriers in the digestive content of fish intended for human consumption in the Atlantic area of Macaronesia. In addition, the aim was to identify sulfhydryl-positive isolates and determine their health implications. To this end, the API and MALDI-TOF MS techniques were used, studying the concordance in the identification between both.

## 2. Materials and Methods

### 2.1. Sample Collection

The present study was conducted between February and May 2023 and included the collection and processing of 59 samples of gastrointestinal contents from various species of fish intended for human consumption and apparently healthy. These were individually frozen at −20 °C. The 59 fish used in this study came from high seas fisheries in FAO area 34, specifically 32 from Mauritania, 13 from Senegal, and 14 from Spain. The fish species included in this study were bluespotted seabream (*Pagrus caeruleostictus*), angolan dentex (*Dentex angolensis*), black moray (*Muraena augusti*), mediterranean moray (*Muraena helena*), white grouper (*Epinephelus aeneus*), pink dentex (*Dentex gibbosus*), redbanded seabream (*Pagrus auriga*), brown moray (*Gymnothorax unicolor*), John Dory (*Zeus faber*), pearly razorfish (*Xyrichtys novacula*), dusky grouper (*Epinephelus marginatus*), and goldblotch grouper (*Epinephelus costae*).

After being caught, they were refrigerated and sent in less than 24–48 h to the University Institute of Animal Health (IUSA) and received through the RASPA project (Atlantic Network for Sanitary Surveillance of Fishery and Aquaculture Products in Macaronesia), originating from the border inspection points of Las Palmas, Barcelona, Algeciras, and Madrid. Upon arrival at IUSA, samples of different organs and tissues were taken, including the gastrointestinal content, which were frozen individually at −20 °C.

### 2.2. Application of the ISO Standard and Bacterial Identification

Each sample was placed in an airtight bag and homogenised in a Stomacher^®^ for approximately 1 min until a uniform mixture was obtained. A swab of each sample was then taken as the starting point for laboratory processing. Once processing was completed, these samples were disposed of using specific containers for the management of this type of waste.

Swabs (VWR, Transport Swabs, Milan, Italy) containing samples obtained from the digestive content of the sampled fish were processed according to ISO 6579-1:2017 [24], looking for carriers of Salmonella spp. and other hydrogen sulphide-positive bacteria resistant to this processing. For this purpose, swabs impregnated with the gastrointestinal contents of each sample were placed in test tubes containing 10 mL of buffered peptone water at room temperature and incubated at 37 °C for 24 h. After this period, 0.1 mL of buffered peptone water was transferred to a Rappaport-Vassiliadis medium (Sigma-Aldrich, St. Louis, MO, USA) and incubated at 41.5 °C for 24 h. The resulting colonies were then reseeded onto two culture media following the instructions of the ISO standard: xylose lysine deoxycholate agar (Sharlau, Spain), as recommended by the standard, and Hektoen enteric agar (Oxoid, UK) as a complementary medium. The plates were incubated at 37 °C for 24 h.

Complementary tests were then carried out, including Gram staining, make a subculture on MacConkey agar (Oxoid, Hampshire, UK), and the oxidase test.

Once it was verified that the strains corresponded to Gram-negative bacilli in pure culture, they were sown on MacConkey agar and incubated at 37 °C for 24 h. This medium allows the growth of Gram-negative bacilli, both lactose fermenters and non-fermenters. A pink colour on this medium indicates that the strains are fermenting, while non-fermenting strains do not acquire this colour [25].

Subsequently, the Gram-negative colonies obtained on MacConkey agar were identified using commercial kits: API 20E (oxidase-negative) or API 20 NE (oxidase-positive) (bioMérieux, Madrid, Spain). The results were interpreted using APIWEB™ software version 1.4.1-3.

Simultaneously, the Gram-negative colonies were replated on an agar base (VWR Chemicals, Leuven, Belgium) with 5% defibrinated sheep blood (Thermo Fisher Scientific/Oxoid). The plates were incubated at 37 °C for 24 to 36 h until adequate growth was observed. The samples were then sent to the Microbiology Service of the Complejo Hospitalario Universitario Insular Materno Infantil (CHUIMI) for identification using the MALDI-TOF MS technique (Bruker Daltonik MALDI Biotyper; Bruker, Billerica, MA, USA).

At the CHUIMI service, the received strains were reseeded. Each was processed as follows. Firstly, colonies were spotted on a metal plate (template) and covered with 0.8 μL of formic acid. This step is called ‘in-target extraction’, which greatly improves identification. After allowing the plate to air dry at room temperature for approximately 10 min, 0.8 µL of matrix solution (α-cyano-3,4-hydroxycinnamic acid) was added to cover the sample, allowing it to dry at room temperature. Finally, the obtained results were entered into a database (operator: tof-user@FLEX-PC, server version: 4.1.100 (PYTH) 188 2020-04-112_10-35-53), and the software provided the identification result (sequence identifier) based on the score values for three evaluation categories.

Reliable identification at species level: SCORE VALUE ≥ 2

Reliable identification at genus level only: SCORE VALUE 1.7–2.0

Unreliable identification: SCORE VALUE < 1.7

### 2.3. Statistical Analysis

For the analysis of the degree of agreement between MALDI-TOF and API, we used the Cohen’s Kappa statistic. The results were categorized using the WinEpi program (http://www.winepi.net/uk, 1 November 2024).

## 3. Results

Of the 59 fish included in this study, 32 came from Mauritania, 14 from Spain, and 13 from Senegal. After processing using the ISO standard, we obtained the development of 47 hydrogen sulphide-positive strains that were isolated from 25 fish from Mauritania, 11 from Spain, and 11 from Senegal. The distribution of fish species by geographical location can be seen in Table 1.

Of the total samples analysed, we obtained 47 strains corresponding to Gram-negative hydrogen sulphide-positive bacteria. All strains grew on MacConkey agar, with 41 of the 47 strains (87.23%) being lactose-positive and oxidase-negative and 6 of the 47 strains (12.77%) being lactose-negative and oxidase-positive.

Of the oxidase-negative strains identified by API 20E, 35 of 47 *Enterobacter cloacae* (74.46%), 3 of 47 *Escherichia hermannii* (6.38%), 1 *Leclercia adecarboxylata* of all 47 isolates (2.12%), 1 *Citrobacter freundii* of all 47 isolates (2.12%), and 1 *Citrobacter koseri/farmeri* of the total of 47 isolates (2.12%) (Table 2) were obtained. *Salmonella* spp. (0.00%) was not isolated.

Of the oxidase-positive strains identified by API 20NE, we obtained 5 of 47 *Shewanella putrefaciens* (10.63%) and 1 *Burkholderia cepacia* of all 47 isolates (2.12%). The most commonly isolated bacterium in our study was *Enterobacter cloacae*. The most frequent biochemical profiles observed in *Enterobacter cloacae* were 3305573 (19 of 47), followed by 3307573 (6 of 47), 3305773 (5 of 47), 3705573 (2 of 47), and one each of 3205573, 3304573, and 3305173. The second most frequently isolated bacterium was *Shewanella putrefaciens*, with the following profiles obtained: 5410114 (3 of 5) and 5411114 (2 of 5). The profiles obtained for the remaining bacteria are listed in Table 3.

Of the total strains identified by MALDI-TOF MS, we obtained the following: 33 of 47 *Enterobacter cloacae* (70.21%); 3 of 47 *Citrobacter freundii* (6.38%); 3 of 47 *Shewanella algae* (6.38%); 2 of 47 *Shewanella indica* (4.25%); 2 of 47 *Escherichia hermannii* (4.25%); 1 each of *Enterobacter bugandensis*, *Enterobacter hormaechei*, and *Enterobacter kobei* (2.12% each); 1 of 47 *Leclercia adecarboxylata* (2.12%) (Table 2).

The results obtained by MALDI-TOF MS are shown in Table 3. The degree of reliability of the MALDI-TOF MS test obtained for each strain is indicated. Of the total strains, 42 of 47 (89.4%) achieved reliable identification to the genus and species level (score value ≥ 2), while 5 of 47 (10.6%) achieved reliable identification to the genus level only (score value between 1.7–2).

In Table 2, we analysed the results obtained from the 47 strains, comparing the identifications obtained after application of the MALDI-TOF MS technique with those obtained by the API technique. To analyse the results, we calculated the concordance percentages between the two identification techniques. Specifically, we calculated the concordance percentages at the genus and species level and also expressed the non-concordant identifications as a percentage, where there was no concordance at either the genus or species level between the MALDI-TOF MS and API identifications.

The concordance results obtained when comparing the 47 strains identified by API and MALDI-TOF MS are as follows: 34 of 47 strains showed concordance between the results of the API technique and the MALDI-TOF MS technique, indicating a 72.34% concordance at the species level. Of these 34, 31 had a score value ≥ 2 and 3 *Enterobacter cloacae* had score values between 1.7–2.0. Additionally, 41 strains showed concordance at the genus level when comparing both techniques, indicating an 87.23% concordance at the genus level. Of these, seven are coincident only at the genus level, five of them with a score value ≥ 2, and two with a score value below 2. Finally, six strains did not present concordance at either the genus or species level between the API and MALDI-TOF MS results, indicating a 12.76% rate of non-concordant identifications; however, all of them had a score value for MALDI TOF-MS ≥ 2.

The results of the tests carried out with MALDI-TOF and API (Table 2 and Table 3) that divide the concordance into four categories according to the score value of the MALDI TOF-MS technique (green, yellow, red and blue) have also been subdivided into four for the calculation of the Kappa coefficient, relating these categories to the score values of the MALDI TOF-MS results.

This categorisation has allowed us to calculate the value of Cohen’s Kappa coefficient as a statistical measure to evaluate the concordance (WinEpi: http://www.winepi.net/uk/, accessed on 1 November 2024, Diagnostic, Test Agreement), thus obtaining the following values:

MALDI-TOF species/API species—31 (score value ≥ 2 and matches species);

MALDI-TOF species/API other species—5 (359, 379, 381, 395, 432) (score value ≥ 2 and matches genus but not species);

MALDI-TOF without species/API other species—6 (371, 377, 388, 3391, 408-2, 447-2) (score value ≥ 2 and does not match genus);

MALDI-TOF without species/API species—5 (384, 385, 386, 430, 445) (score value ≤ 2 and matches genus but three match in species);

Kappa coefficient—0.407;

Observed proportion of agreement (concordance rate)—0.7872 (78.72%);

Expected agreement ratio—0.6415 (54.15%);

Kappa (95–99.5% confidence level)—0.407;

Evaluation—Kappa between 0.41 and 0.60 = moderate agreement.

Specifically, 35 of 47 (74.46%) of the strains identified by the API technique were identified as *Enterobacter cloacae*. Of these, 31 of 35 strains showed concordance at the genus and species level with respect to the MALDI-TOF MS results, resulting in an 88.57% concordance at the genus and species level for this bacterium. Additionally, 34 of 35 *Enterobacter* strains presented concordance at the genus level only, representing a 97.14% concordance at the genus level. One strain did not present agreement at either the genus or species level with the MALDI-TOF MS results, representing a 2.86% rate of non-concordant identifications; this was identified by MALDI-TOF MS as *Citrobacter freundii*.

*Escherichia hermannii* was identified in 3 of 47 (6.38%) strains, with one of three (33.3%) matching at the genus and species level. Two of three (66.6%) did not match at the genus level with MALDI-TOF MS; this was identified by MALDI-TOF MS as *Enterobacter cloacae*.

*Citrobacter freundii* and *Leclercia adecarboxylata* were each identified once using the API technique and showed 100% concordance at the genus and species level with the MALDI-TOF MS results. *Citrobacter koseri/farmeri* was identified once using the API technique and showed 100% non-concordant identification with the MALDI-TOF MS results; MALDI-TOF MS identified this as *Escherichia hermannii*.

The concordance results obtained when comparing all the Gram-negative oxidase-negative bacilli identified by API 20E with those identified by the MALDI-TOF MS technique showed that 37 of 41 strains (90.24%) were correlated at the genus level, 34 of 41 (82.92%) were correlated at the species level, and 4 of 41 (9.75%) were not correlated at either the genus or species level.

Regarding *Shewanella putrefaciens*, we obtained 5 of 47 (10.63%) identifications using the API technique. When comparing these results with those obtained by MALDI-TOF MS, we found that four of five showed concordance at the genus level only, resulting in an 80% concordance at genus level; one of five (20%) did not coincide at the genus level; MALDI-TOF MS identified this as *Citrobacter freundii*.

The same was true for *Burkholderia cepacia*, which was identified once using the API technique and showed 100% non-concordant identification with the MALDI-TOF MS results; MALDI-TOF MS identified this as *Shewanella indica*.

The concordance results obtained when comparing the bacilli identified by API 20NE (6 of 6) with those identified by the MALDI-TOF MS technique showed that four of six strains (66.66%) were correlated at the genus level, none (0.00%) were correlated at the species level, and two of six (33.33%) were not correlated at either the genus or species level.

**Figure 1:**
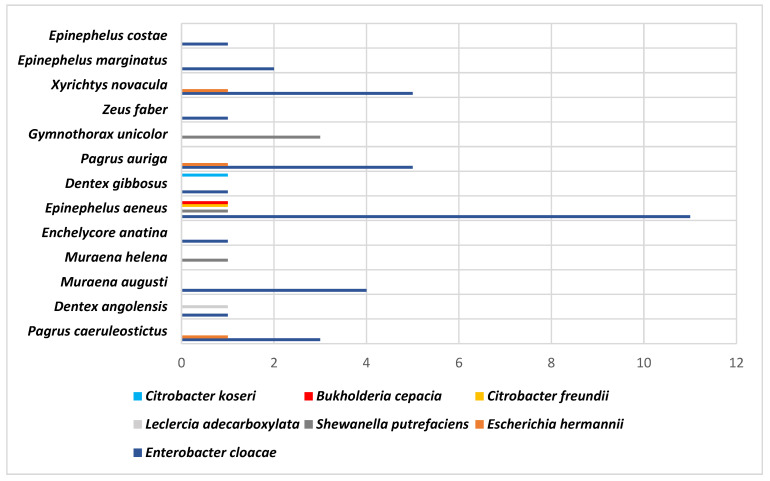
Identifications obtained using the API technique according to fish species.

**Figure 2:**
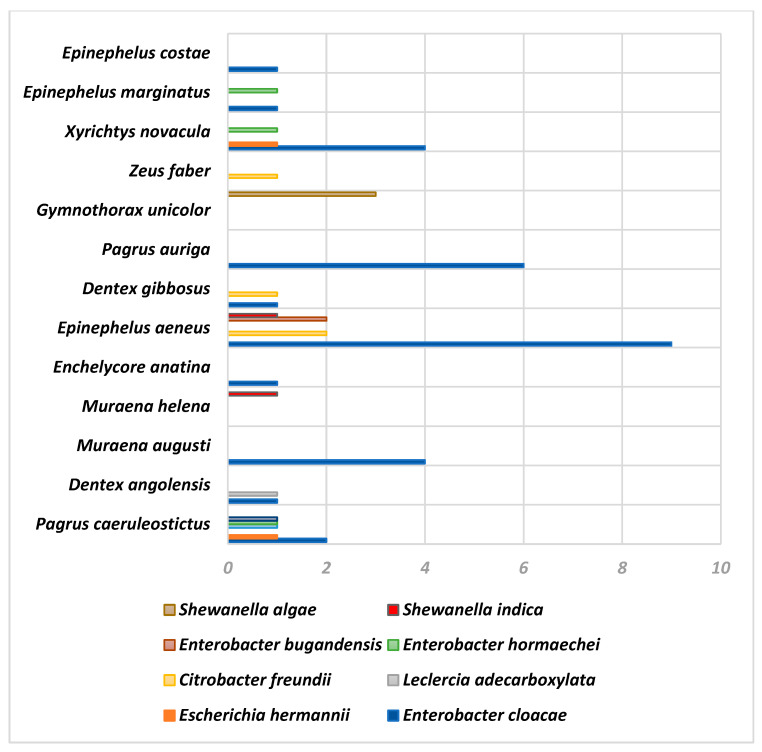
Identifications obtained using the MALDI-TOF MS technique according to fish species.

Finally, the following figures show the results obtained using API (Figure 1) and MALDI-TOF MS (Figure 2) and their relationship with the fish species and the strains obtained.

## 4. Discussion

*Salmonella* is an uncommon bacterium in fish, and its occurrence depends on the quality of the water and the aquatic environment [2]. Likewise, we did not obtain any isolates of this bacterium in our samples, consistent with findings by Herrera et al. (2006) in León, Spain [26]. Similar results have also been reported by other authors [27].

Fish can carry bacteria on the surface of their bodies and/or in their guts and can act as carriers of bacteria. Human salmonellosis linked to the consumption of infected fish is usually due to *Salmonella typhimurium* and *Salmonella enteritidis*. The persistence of *Salmonella* in the digestive tract of fish and its presence in faeces is attributed to environmental contamination and bacterial spread [28]. In humans, *Salmonella* spp. can cause clinical complications such as sepsis, abdominal pain, diarrhoea, and vomiting [29]. *Salmonella* can be found on the skin, gills, and intestines of infected fish, and it has been shown that this bacterium can survive the processing of smoked fish and be transmitted to humans through consumption [30]. Similarly, Novotny et al. (2004) [31] and Bonyadian et al. (2014) [32] reported *Salmonella typhimurium* infection due to the consumption of imported dried fish. Other *Salmonella* isolates have also been documented in freshwater and saltwater fish, raw shellfish, bivalve molluscs from contaminated growing areas, and even smoked fish [33].

Before discussing the different bacteria isolated in our study, we must point out that all the isolates described below showed tolerance to high temperatures. All of them withstood a temperature of 41.5 °C, since they were isolated after applying the ISO standard, and persisted in the marine environment at temperatures of 18 °C to 24 °C, even withstanding sea salinity. These isolates presented limitations, since conventional media or other resources for the isolation of other bacteria were not used. We have not found previous references in the literature describing isolates like those obtained in this study.

Regarding the API/MALDI-TOF MS identification of the 47 bacterial strains obtained, all of which were Gram-negative, we observed that 34 of them showed agreement between both techniques, presenting a high concordance in species level (72.34%). This concordance rate is somewhat lower than the results obtained by Ferreira et al. (2010) [34], who reported an API 20E/MALDI-TOF MS concordance of 87.8%.

In the present study, when considering only the oxidase-negative bacteria identified by API 20E, the concordance in genus and species with MALDI-TOF MS rose to 82.92%, which is very close to the value obtained by Ferreira et al. (2010) [34]. The genus concordance reported by these authors was 97.8%, slightly higher than our overall result of 87.23% for all Gram-negative strains. However, when focusing solely on the oxidase-negative bacteria identified by API 20E in our study, the genus concordance with MALDI-TOF MS increased to 90.24%, approaching the results reported by Ferreira et al. (2010) [34].

It was confirmed in the present study that some species of *Enterobacter*, such as *Enterobacter bugandensis*, *Enterobacter hormaechei*, and *Enterobacter kobei*, are not included in the API 20E database (bioMérieux). These were identified in our study using the MALDI-TOF MS technique, but they had all previously been identified as *Enterobacter cloacae* through API 20E. We found no relationship between the biochemical profiles (API 20E) of these species and those identified as *Enterobacter cloacae*, with the most common profiles being 3305573 for *Enterobacter hormaechei* and *Enterobacter kobei* and 3307573 for *Enterobacter bugandensis*.

Uchida et al. (2020) [35] pointed out that the accurate identification of *Enterobacter species* is problematic. They also noted that biochemical identification methods, including the API 20E test, which is frequently used, can give conflicting results because of the limited discriminatory power of its reference database.

The identification of these species using a PCR could be considered in the present study. However, Uchida et al. (2020) [35] stated that although 16S rRNA gene sequencing is one of the gold standards for bacterial identification, it often cannot clearly identify *Enterobacter* species because its results do not agree well with taxonomic classifications at the species level.

In the case of *Escherichia hermannii*, we obtained two non-concordant identifications out of three, with the strains being identified by MALDI-TOF MS as *Enterobacter cloacae*. Given this discordance, we believe that a PCR should be performed to discern the correct identification, as suggested by Méndez-Álvarez and Pérez-Roth (2004) [36]. However, according to Uchida et al. (2020) [35], even a PCR might not guarantee correct identification.

For *Leclercia adecarboxylata*, there was a 100% genus–species concordance between the two techniques [37].

On the other hand, in the case of *Shewanella*, we found a low concordance when referring to species. Of the five strains in the *Shewanella putrefaciens* group identified by the API 20NE technique, three were identified by the MALDI-TOF MS technique as *Shewanella algae*, one as *Shewanella indica*, and another as *Citrobacter freundii*.

Moreover, another strain identified as *Shewanella indica* by MALDI-TOF MS was identified as *Burkholderia cepacia* by API 20NE. Notably, *Shewanella algae* and *Shewanella indica* are not included in the API 20NE databases [38]. These authors considered MALDI-TOF MS an ideal technique for identifying species belonging to the genus *Shewanella*, which suggests that the correct identification could be provided by MALDI-TOF MS, especially considering that the API databases may not be up to date.

Furthermore, Ferreira et al. (2010) [34] noted that non-concordant identifications (genus-level errors] often occur in relatively rare genera and species, such as *Raoultella*, or in species where identification by classical methods can be challenging and not reliable, such as *Acinetobacter lwoffii* or *Pseudomonas putida*. In the present study, this occurred with the identification of *Burkholderia cepacia*, which was identified by MALDI-TOF MS as *Shewanella indica*.

Overall, the total percentage of non-corresponding identifications was 12.76% (6 of 47), with a discrepancy of agreement in API 20E of 9.75% (4 of 41) and 33.33% in API 20NE (two of six). Quantitatively, the largest discrepancies were detected when comparing the identifications obtained by API 20NE and MALDI-TOF MS.

Based on the results obtained, we believe that MALDI-TOF MS has several characteristics that make it more advantageous than conventional biochemical methods, particularly for the sophisticated identification of Gram-negative oxidase-positive bacilli [39]. Additionally, this technique shortens the identification process by approximately 24 h. Traditional biochemical identification methods, such as the bioMérieux API system, require an additional 24 to 48 h to provide results because they are based on evaluating the metabolic activity of microorganisms using galleries [40].

MALDI-TOF MS also offers other advantages. Ferreira et al. (2010) [34] demonstrated that it is a highly accurate technique. Technological advancements with MALDI-TOF MS enable the comparison of results with an extensive database, assigning a degree of reliability (score value) to each identification of the results achieved. Although the initial investment in the equipment can be substantial, consumable costs are minimal because the metal extension and readout plates can be reused [34]. Additionally, due to the stability of ribosomal proteins, the MALDI-TOF MS technique can effectively identify bacteria regardless of the type of culture medium and incubation time significantly affecting the results [41]. Moreover, using MALDI-TOF MS for the rapid identification of bacteria from positive blood cultures (without waiting for colony formation) is crucial, particularly in cases of sepsis, for the early administration of appropriate treatment to patients [39]. Operating costs and consumables are significantly lower than with traditional methods such as API [19]. This is reflected in the study by Seng et al. (2009) [42], which showed that the cost of identifying bacteria using MALDI-TOF MS was only 17% to 32% of the cost of traditional identification methods. This equated to approximately €1.43 per sample with MALDI-TOF MS, compared with €4.60 to €8.23 per sample using conventional methods. Although the initial investment in MALDI-TOF MS equipment can be considerable, an economic analysis conducted by García et al. (2012) [41] suggests that the investment is recovered within 4 years due to savings in laboratory materials, reduced labour time, and fewer repetitions in microbiological identification with traditional manual methods.

Finally, MALDI-TOF MS has been used to identify a wide range of microorganisms, including bacteria, fungi, and viruses [43]. Thanks to its ability to rapidly characterise these organisms, MALDI-TOF MS has potential applications in medical diagnosis, biological defence, environmental monitoring, and quality control in the food industry [19].

However, MALDI-TOF mass spectrometry has certain limitations, particularly in the identification of pathogens in clinical samples [39] and in the identification of certain filamentous and yeast-like fungi. The accuracy of MADI-TOF mass spectrometry results is directly dependent on existing master spectral profiles (MSPs). However, urine and other infected fluids may be suitable samples for direct identification with MALDI-TOF mass spectrometry [40].

In light of the above, we believe that the choice of using MALDI-TOF MS or conventional bacterial identification methods will depend on the specific laboratory context and diagnostic needs. As demonstrated, the MALDI-TOF MS technique offers significant advantages in terms of speed, accuracy, and long-term operational costs. It can be considered the ideal bacterial identification technique in clinical settings where early and reliable identification is crucial for diagnosis and treatment. Conversely, conventional methods remain important and valuable in environments where technology and financial resources are limited.

In this study, bacteria of health significance were successfully isolated. The genus *Enterobacter* accounted for 36 out of 47 strains (76.5%) identified in the analysis. *Enterobacter cloacae* was the most common microorganism present, accounting for 70.21% (33 of 47) of the total isolates by MALDI-TOF MS. In addition to *Enterobacter cloacae*, we also identified *Enterobacter bugandensis*, *Enterobacter hormaechei*, and *Enterobacter kobei*, each representing 2.12% (1 of 47) of the total isolates by MALDI-TOF MS. These bacteria are Gram-negative and belong to the *Enterobacteriaceae* family. They are considered opportunistic pathogens in humans, mainly affecting patients with compromised immune systems, and are associated with infections such as neonatal sepsis. They are common in nosocomial infections and can cause bacteraemia, wound, respiratory tract, intestinal tract, and urinary tract infections. They are found in nature and in the normal microbiota of the gastrointestinal tract in humans and animals. However, *Enterobacter cloacae* is frequently detected in wastewater samples. Currently, *Enterobacter cloacae* and *Enterobacter hormaechei* are the most frequently isolated *Enterobacteriaceae* species in intensive care patients. However, *Enterobacter bugandensis* is highly virulent and possibly the most pathogenic species of the genus [44,45]. To prevent these infections, antibiotics have been widely used in aquaculture. However, the overuse of these drugs has led to the development of resistant strains, posing a serious risk to consumers [46].

Another bacterium identified by MALDI-TOF MS was *Citrobacter freundii*, which was isolated twice, accounting for 4.25% (2 of 47) of the total isolates. Although *Citrobacter koseri/farmeri* was found only once in the API identification, it was not present in the MALDI-TOF MS results. Both species are Gram-negative bacteria and belong to the *Enterobacteriaceae* family. They are commonly found in water, soil, food, and the intestinal tract of humans and animals. Both bacteria are considered pathogenic for humans and can cause urinary tract infections, respiratory tract infections, intra-abdominal infections, wound infections, blood infections, and central nervous system infections, particularly in hospitalised patients with multiple comorbidities. *Citrobacter freundii* and *Citrobacter koseri* are the species most associated with infections in humans [47]. *Citrobacter koseri* is a known cause of meningitis and brain abscesses in neonates [48]. More than 10 years ago, reports of abscesses caused by *Citrobacter koseri* in adults were rare. However, there has been an increase in *Citrobacter koseri* infections in adults, even in those without pre-existing conditions [49]. Like other enterobacteria, some species of *Citrobacter* have developed resistance to many antimicrobial agents, including beta-lactams, posing a significant challenge in managing the infections they cause [47].

We also identified *Escherichia hermannii* twice, representing 4.25% (2 of 47) of the total isolates by MALDI-TOF MS. This Gram-negative bacterium belongs to the *Enterobacteriaceae* family. Although it is an infrequent agent in human infections, it is generally considered a co-infectant in infections with other pathogens. However, there is evidence to suggest that this bacterium has pathogenic potential, causing infections even in immunocompetent individuals [50]. It has been isolated from wounds, peritonitis, conjunctivitis, blood, cerebrospinal fluid, and urine. The most frequent infections occur in the bloodstream, urinary tract, and central nervous system [51].

*Leclercia adecarboxylata* was identified once, representing 2.12% (1 of 47) of the total isolates by MALDI-TOF MS. *Leclercia adecarboxylata* is a Gram-negative bacterium that belongs to the *Enterobacteriaceae* family. It has been found in food, water, and environmental or animal sources. Additionally, it is an emerging opportunistic pathogen in humans, primarily in immunocompromised individuals, although it has also been observed in polymicrobial infections in immunocompetent individuals. *Leclercia adecarboxylata* has been detected in blood, faeces, sputum, urine, and wound pus [37].

Finally, of the six oxidase-positive strains, MALDI-TOF MS identified five strains within the *Shewanella* genus, classified as *Shewanella algae* and *Shewanella indica*. Of these, four of six had previously been identified using the API technique as *Shewanella putrefaciens*. These bacteria are part of the aquatic environment, both in freshwater and saltwater, but have also been isolated from soil and animal products [52]. Contamination of food with *Shewanella* spp. leads to food spoilage, especially in seafood. *Shewanella* is an opportunistic pathogen, and exposure to these bacteria through activities in contaminated marine environments or ingestion of contaminated food can cause various skin and soft tissue infections, systemic diseases, hepatobiliary infections, and otitis media, among others [53,54]. Additionally, *Shewanella algae* has been recognised as a human pathogen, and *Shewanella indica* was recently identified in an abscess in a Bryde’s whale. *Shewanella indica* infections have not yet been reported in humans or animals [55]. The results obtained in this genus are significant since some research indicates that *Shewanella algae* and *Shewanella putrefaciens* are opportunistic pathogens in various marine species. In China and Taiwan, strains of *Shewanella algae* have been isolated as responsible for mortality in farmed abalone and for causing ulcerative disease in sea bass (*Sciaenops ocellata*). *Shewanella putrefaciens* has also been identified as a virulent bacterium in juvenile freshwater zebra mussels. *Shewanella marisflavi* has been reported to be highly pathogenic to sea cucumbers. These findings could be relevant and have a health impact in the marine environment of the Macaronesia area [56].

Finally, according to Doern (2013) [57], when the laboratory performing the MALDI TOF-MS test has validated its results for years with a large number of isolates for the identified species, as has been the case in our CHUIMI laboratory, verification by another test is not considered necessary if the score value is ≥2, nor is verification by the gold standard test for bacterial identification, which is the detection of the 16S rRNA gene, required. The infrastructure required to implement this technique is highly expensive, and many laboratories lack it, as is the case in ours. Likewise, this review article [57] states that when MALDI TOF-MS results are compared with those obtained with other biochemical (API) or genetic techniques approved by the FDA (Food and Drug Administration), and their identifications are coincident, the MALDI TOF-MS identifications will be considered valid. Six strains in this study were identified as API/MALDI TOF-MS mismatches (12.76%), but the MALDI TOF-MS score values were all ≥ 2. Therefore, as we mentioned above when the laboratory has validated its results for years, these identifications do not need to be validated by other techniques. With all the above, our strains are identified at the species level, except for two *Shewanella* spp. that are verified only at the genus level. Although the MALDI-TOF and API databases are validated mainly for human isolates, their high accuracy supports their use in this study, given that the bacteria isolated in this work have an impact on public health and are therefore contained in these databases.

## 5. Conclusions

In the present study, the MALDI-TOF MS technique has proven to be the method of choice for identifying bacteria that are difficult to diagnose and have significant health implications, such as *Shewanella* spp., *Enterobacter bugandensis*, *Enterobacter hormaechei*, and *Enterobacter kobei*. API commercial galleries have outdated databases, but they remain useful for identifying common bacteria that have not undergone recent reclassifications. Most of the bacteria we isolated in our study probably originate from wastewater and might pose a public health risk. However, we believe it is important to highlight the isolation of *Shewanella* as the only bacteria of marine origin identified, which could directly affect the health of animals living in that environment.

## Figures and Tables

**Table 1:** Fish species and geographical location.

ESPECIES	SPAIN	MAURITANIA	SENEGAL
***Pagrus caeruleostictus***	0	2	2
***Dentex angolensis***	0	2	0
***Muraena augusti***	4	0	0
***Muraena helena***	1	0	0
***Enchelycore anatina***	1	0	0
***Epinephelus aeneus***	0	14	0
***Dentex gibbosus***	0	2	0
***Pagrus auriga***	1	4	1
***Gymnothorax unicolor***	3	0	0
***Zeus faber***	0	0	1
***Xyrichtys novacula***	0	0	6
***Epinephelus marginatus***	0	1	1
***Epinephelus costae***	0	1	0
***TOTAL***	11	25	11

**Table 2:** Comparison of the identification of 47 bacteria using the API and MALDI-TOF MS techniques.

API Identifications (n° of Strain)	Concordance in the Species %	Concordance in the Genera %	No Concordance Identifications %	MALDI-TOF MS Identifications (n° of Strain)
*Enterobacter cloacae* 74.46% (35/47)	100	100	0	*Enterobacter cloacae* (28)
	100	100	0	*Enterobacter cloacae* (3)
	0	100	0	*Enterobacter bugandensis* (1)
	0	100	0	*Enterobacter hormaechei* (1)
	0	100	0	*Enterobacter kobei* (1)
	0	0	100	*Citrobacter freundii* (1)
*Escherichia hermannii* 6.38% (3/47)	100	100	0	*Escherichia hermannii* (1)
	0	0	100	*Enterobacter cloacae* (2)
*Citrobacter freundii* 2.12% (1/47)	100	100	0	*Citrobacter freundii* (1)
*Citrobacter koseri/farmeri* 2.12% (1/47)	0	0	100	*Escherichia hermannii* (1)
*Leclercia adecarboxylata* 2.12% (1/47)	100	100	0	*Leclercia adecarboxylata* (1)
*Shewanella putrefaciens* 10.63% (5/47)	0	100	0	*Shewanella indica* (1)
	0	100	0	*Shewanella algae* (1)
	0	100	0	*Shewanella algae* (2)
	0	0	100	*Citrobacter freundii* (1)
*Burkholderia cepacia* 2.12% (1/47)	0	0	100	*Shewanella indica* (1)
TOTAL (47)	72.34%	87.23%	12.76%	

Note: Four colours were used to improve visualisation and organisation. Green indicates agreement at the genus and species level between the identification made with the API technique and the MALDI-TOF MS technique (score value ≥ 2). Yellow, score value ≥ 2 and matches genus but not species. Red, score value ≥ 2 and does not match genus, and blue score value ≤ 2 and matches genus but three match in species.

**Table 3:** API biochemical profiles and degree of reliability of the MALDI-TOF MS test obtained from the 47 strains.

Code Fishes RASPA Project	API Profiles	API Identifications	MALDI-TOF MS Identifications	Value of MALDI-TOF MS
326	1144113	*Escherichia hermannii*	*Escherichia hermannii*	2.28
340	0044553	*Leclercia adecarboxylata*	*Leclercia adecarboxylata*	2.41
354	3305773	*Enterobacter cloacae*	*Enterobacter cloacae*	2.31
355	3305773	*Enterobacter cloacae*	*Enterobacter cloacae*	2.33
356	3305773	*Enterobacter cloacae*	*Enterobacter cloacae*	2.32
357	3305773	*Enterobacter cloacae*	*Enterobacter cloacae*	2.34
359	3307573	*Enterobacter cloacae*	*Enterobacter bugandensis*	2.12
360	3205573	*Enterobacter cloacae*	*Enterobacter cloacae*	2.19
366	3304573	*Enterobacter cloacae*	*Enterobacter cloacae*	2.24
371	1744573	*Citrobacter koseri/farmeri*	*Escherichia hermannii*	2.45
375	3705573	*Enterobacter cloacae*	*Enterobacter cloacae*	2.17
376	3305573	*Enterobacter cloacae*	*Enterobacter cloacae*	2.24
377	1144133	*Escherichia hermannii*	*Enterobacter cloacae*	2.23
379	5410114	*Shewanella putrefaciens* group	*Shewanella indica*	2.04
381	5411114	*Shewanella putrefaciens* group	*Shewanella algae*	2.02
384	5411174	*Shewanella putrefaciens* group	*Shewanella algae*	1.91
385	5410114	*Shewanella putrefaciens* group	*Shewanella algae*	1.74
386	3305573	*Enterobacter cloacae*	*Enterobacter cloacae*	1.94
388	3705573	*Enterobacter cloacae*	*Citrobacter freundii*	2.26
390	3305173	*Enterobacter cloacae*	*Enterobacter cloacae*	2.19
391	1144133	*Escherichia hermannii*	*Enterobacter cloacae*	2.28
392	3305573	*Enterobacter cloacae*	*Enterobacter cloacae*	2.05
393	3305573	*Enterobacter cloacae*	*Enterobacter cloacae*	2.22
394	3305573	*Enterobacter cloacae*	*Enterobacter cloacae*	2.14
395	3305573	*Enterobacter cloacae*	*Enterobacter hormaechei*	2.16
404	3305573	*Enterobacter cloacae*	*Enterobacter cloacae*	2.16
405	3305573	*Enterobacter cloacae*	*Enterobacter cloacae*	2.20
407	3305573	*Enterobacter cloacae*	*Enterobacter cloacae*	2.28
408-2	5410114	*Shewanella putrefaciens* group	*Citrobacter freundii*	2.10
408-1	3307573	*Enterobacter cloacae*	*Enterobacter cloacae*	2.26
414	3305573	*Enterobacter cloacae*	*Enterobacter cloacae*	2.04
417	3305573	*Enterobacter cloacae*	*Enterobacter cloacae*	2.16
418	3305573	*Enterobacter cloacae*	*Enterobacter cloacae*	2.16
426	3305573	*Enterobacter cloacae*	*Enterobacter cloacae*	2.11
427	3305573	*Enterobacter cloacae*	*Enterobacter cloacae*	2.22
429	3305573	*Enterobacter cloacae*	*Enterobacter cloacae*	2.18
430	3307573	*Enterobacter cloacae*	*Enterobacter cloacae*	1.77
431	3307573	*Enterobacter cloacae*	*Enterobacter cloacae*	2.26
432	3305573	*Enterobacter cloacae*	*Enterobacter kobei*	2.16
435	3305573	*Enterobacter cloacae*	*Enterobacter cloacae*	2.23
444	3305573	*Enterobacter cloacae*	*Enterobacter cloacae*	2.38
445	3307573	*Enterobacter cloacae*	*Enterobacter cloacae*	1.96
446	3305573	*Enterobacter cloacae*	*Enterobacter cloacae*	2.06
447-1	3307573	*Enterobacter cloacae*	*Enterobacter cloacae*	2.34
447-2	1577757	*Burkholderia cepacia*	*Shewanella indica*	2.10
448-1	1604773	*Citrobacter freundii*	*Citrobacter freundii*	2.35
448-2	3305773	*Enterobacter cloacae*	*Enterobacter cloacae*	2.13

Note: Four colours were used to improve visualisation and organisation. Green indicates agreement at the genus and species level between the identification made with the API technique and the MALDI-TOF MS technique (score value ≥ 2). Yellow, score value ≥ 2 and matches genus but not species. Red, score value ≥ 2 and does not match genus, and blue score value ≤ 2 and matches genus but three match in species.

## Data Availability

The data presented in this study are contained within this article.
